# The prevalence and density of asymptomatic *Plasmodium falciparum* infections among children and adults in three communities of western Kenya

**DOI:** 10.1186/s12936-021-03905-w

**Published:** 2021-09-17

**Authors:** Christina Salgado, George Ayodo, Michael D. Macklin, Meetha P. Gould, Srinivas Nallandhighal, Eliud O. Odhiambo, Andrew Obala, Wendy Prudhomme O’Meara, Chandy C. John, Tuan M. Tran

**Affiliations:** 1 Division of Infectious Diseases, Department of Medicine, Indiana University School of Medicine, Indianapolis, IN USA; 2 Ryan White Center for Pediatric Infectious Diseases and Global Health, Department of Pediatrics, Indiana University School of Medicine, Indianapolis, IN USA; 3 Center for Global Health Research, Kenya Medical Research Institute, Kisumu, Kenya; 4 Jaramogi Oginga Odinga University of Science and Technology, Bondo, Kenya; 5 Reserve Analytics, LLP, Cleveland, OH USA; 6 School of Medicine, Moi University College of Health Sciences, Eldoret, Kenya; 7 Duke Global Health Institute, Duke University, Durham, NC USA

**Keywords:** Malaria, *Plasmodium falciparum*, Asymptomatic infection, Gametocytes, PIESP2, Cross-sectional study, Parasitemia, Sub-microscopic, Infectious reservoir

## Abstract

**Background:**

Further reductions in malaria incidence as more countries approach malaria elimination require the identification and treatment of asymptomatic individuals who carry mosquito-infective *Plasmodium* gametocytes that are responsible for furthering malaria transmission. Assessing the relationship between total parasitaemia and gametocytaemia in field surveys can provide insight as to whether detection of low-density, asymptomatic *Plasmodium falciparum* infections with sensitive molecular methods can adequately detect the majority of infected individuals who are potentially capable of onward transmission.

**Methods:**

In a cross-sectional survey of 1354 healthy children and adults in three communities in western Kenya across a gradient of malaria transmission (Ajigo, Webuye, and Kapsisywa–Kipsamoite), asymptomatic *P. falciparum* infections were screened by rapid diagnostic tests, blood smear, and quantitative PCR of dried blood spots targeting the *varATS* gene in genomic DNA. A multiplex quantitative reverse-transcriptase PCR assay targeting female and male gametocyte genes (*pfs25*, *pfs230p*), a gene with a transcriptional pattern restricted to asexual blood stages (*piesp2*), and human *GAPDH* was also developed to determine total parasite and gametocyte densities among parasitaemic individuals.

**Results:**

The prevalence of *varATS*-detectable asymptomatic infections was greatest in Ajigo (42%), followed by Webuye (10%). Only two infections were detected in Kapsisywa. No infections were detected in Kipsamoite. Across all communities, children aged 11–15 years account for the greatest proportion total and sub-microscopic asymptomatic infections. In younger age groups, the majority of infections were detectable by microscopy, while 68% of asymptomatically infected adults (> 21 years old) had sub-microscopic parasitaemia. *Piesp2*-derived parasite densities correlated poorly with microscopy-determined parasite densities in patent infections relative to *varATS*-based detection. In general, both male and female gametocytaemia increased with increasing *varATS*-derived total parasitaemia. A substantial proportion (41.7%) of individuals with potential for onward transmission had qPCR-estimated parasite densities below the limit of microscopic detection, but above the detectable limit of *varATS* qPCR.

**Conclusions:**

This assessment of parasitaemia and gametocytaemia in three communities with different transmission intensities revealed evidence of a substantial sub-patent infectious reservoir among asymptomatic carriers of *P. falciparum*. Experimental studies are needed to definitively determine whether the low-density infections in communities such as Ajigo and Webuye contribute significantly to malaria transmission.

**Supplementary Information:**

The online version contains supplementary material available at 10.1186/s12936-021-03905-w.

## Background

Malaria remains a global public health burden with 229 million cases worldwide in 2019 [1]. Transmission of malaria requires that sexual-stage *Plasmodium* parasites, gametocytes, present in the blood of infected humans be ingested by female *Anopheles* mosquitoes during feeding. Strategies that combine effective control of the mosquito vector through use of insecticide-treated nets (ITNs) and indoor residual spraying alongside rapid diagnosis and effective treatment of malaria with artemisinin-based combination therapy (ACT) have reduced the prevalence of *Plasmodium falciparum* infection and the incidence of clinical malaria in endemic areas of Africa since 2000, albeit at a slower rate in recent years [1]. Further reductions in malaria incidence as more countries approach malaria elimination would require the identification and treatment of asymptomatic individuals, who carry mosquito-infective gametocytes that are responsible for furthering malaria transmission [2].

Detection of asymptomatically infected individuals has been a major challenge given that individuals residing in areas of high-transmission intensity often carry parasitaemia at densities below the detection limits of accessible field diagnostics, which currently includes microscopy and rapid diagnostic tests (RDTs) [3]. Moreover, the proportion of low-density infections among all malaria infections in a community increases with decreasing malaria transmission [2], suggesting that more sensitive diagnostics are required for detecting parasitaemia among individuals in low-transmission settings [4]. Several studies have examined whether low-density infections contribute to onward transmission using mosquito feeding assays [5–11]. A recent meta-analysis of eight such studies estimated that individuals with sub-patent parasitaemia were approximately one-third as infectious to mosquitoes as individuals with blood-smear positive infections [4]. In general, gametocyte density directly correlates with mosquito infectivity and thus transmission, with infections with parasite densities below the limit of detection of conventional molecular diagnostics being unlikely to contribute significantly to transmission [12]. Assessing the relationship between total parasitaemia and gametocytaemia in field surveys can provide insight as to whether detection of low-density, asymptomatic *P. falciparum* infections using sensitive molecular methods can identify the majority of infected individuals who are potentially capable of onward transmission.

In this study, quantitative molecular assays were used to determine the prevalence and density of asymptomatic *P. falciparum* infections among children and adults in three communities of western Kenya that differed in transmission intensities. To better estimate the relationship between asexual parasite densities and gametocyte densities, a multiplex quantitative reverse-transcriptase polymerase chain reaction (PCR) assay for detecting asexual stage-specific, female gametocyte-specific, and male gametocyte-specific genes in a single blood sample was developed and evaluated. Results were compared to microscopy and an established quantitative PCR-based diagnostic assay.

## Methods

### Ethics approval and consent to participate

The study was reviewed and approved by the Kenya Medical Research Institute Scientific and Ethics Review Unit and the Indiana University Institutional Review Board. Written informed consent was obtained from a parent or guardian of participants who were minors and from adult participants. Minors aged 13–17 years provided their own written informed assent, accompanied by written consent of a parent or guardian.

### Study sites and study participants

The study was conducted from August to September 2016 at three sites in western Kenya that differed in malaria transmission intensity. This time period was specifically chosen as it was one month after the primary malaria transmission peak but before the secondary peak for all three sites and thus increased the likelihood of recruiting individuals who had not experience symptomatic malaria within the last 30 days. Ajigo is located in the lowland area of Siaya County, where malaria transmission is intense and perennial, but with a seasonal peak from May to July [13, 14]. The Webuye township is in Bungoma County, which exhibits moderate, perennial transmission with a primary seasonal peak in May to June and a smaller, second peak in October [15–17]. Kapsisywa and Kipsamoite are two adjacent highland communities in Nandi County with low and unstable malaria transmission. Transmission for these two sites is highly variable, with a typical primary peak in May to July and an occasional smaller peak in November to December that varies from year to year [18, 19]. Intended recruitment targets were 200 participants in each of the moderate-to-high-transmission sites (the Matulo sublocation of Webuye and Ajigo) and 450 participants in each of the low-transmission sites (Kapsisywa and Kipsamoite). Sample sizes of 200, 450, and 900 participants provided 95% confidence of detecting within 20% of the true proportion of asymptomatic infections if the unknown prevalence was estimated to be 43%, 25%, and 14%, respectively. Healthy participants aged 1 to 85 years were recruited from a randomized community census of households for each site in an age-stratified manner to ensure adequate sample sizes for each age group, with a maximum of two participants for any household. Recruitment and enrollment occurred over a -week period. A brief questionnaire that included gender, age, recent travel history within the last month, recent use of ITNs, and relevant medical history was administered. Exclusion criteria at enrollment were axillary temperature ≥ 37.5 °C or history of fever, acute symptomatic illness, underlying chronic disease, malaria in the last 30 days, use of anti-malarial or immunosuppressive medications in the last 30 days, or pregnancy.

### Blood collection

Drops of blood were collected by fingerprick for Paracheck Pf RDT (Orchid Biomedical Systems), which detects the presence of *P. falciparum* histidine-rich protein 2 in blood specimens; whole-blood ribonucleic acid (RNA); thick and thin blood smears; and dried blood spots (DBS) on filter paper (903 Protein Saver; Whatman). Individuals who tested positive for asymptomatic *P. falciparum* infection by RDT were treated at the point-of-care using the standard regimen recommended by the Ministry of Health in Kenya. For whole-blood RNA, 200 μl of peripheral fingerprick blood was collected using capillary blood collection tubes containing *Tris*-ethylenediaminetetraacetic acid (EDTA; Microvette CB300 K2E; Sarstedt) and transferred immediately in cryotubes pre-filled with 400 μl Tempus solution (Applied Biosystems). Filled sample tubes were agitated vigorously per the manufacturer’s instructions and stored at − 80 °C within 24 h of collection until use.

### Microscopy

Giemsa-stained blood smears were examined for the presence of asexual parasites in 200 fields using the 100× oil immersion objective lens by two trained microscopists. Independent verification was performed by a third reader for samples that were qualitatively discordant for positivity between the first two microscopists. For positive samples, the number of asexual parasites per 200 leukocytes was multiplied by 40 to convert to parasites per μl, assuming an average leukocyte count of 8000 leukocytes per μl of blood. The mean parasite density from the two concordant microscopists were used for analysis.

### Parasite culture

To produce parasite genomic deoxyribonucleic acid (gDNA) for use in standard curves for parasite density determination, *P. falciparum* 3D7 parasites [Malaria Research and Reference Reagent Resource Center (MR4), BEI Resources] were cultured in vitro using standard techniques [20] with two rounds of synchronization by sorbitol treatment to achieve a high parasitaemia. Ring and early trophozoite stage *P. falciparum* parasites were tenfold serially diluted in whole blood of an uninfected North American donor to obtain a final density of 440,000 down to 0.44 parasites/µl and spotted on 903 Protein Saver cards. To produce parasite RNA for use in standard curves for gametocyte density estimates, *P. falciparum* NF54 (MR4) in vitro cultures were enriched for gametocytes by decreasing asexual parasitaemia [17]. Total gametocytes (without differentiating for sex) were counted and tenfold serially diluted in whole blood to obtain a final density of 2580 down to 0.00258 gametocytes/μl immediately prior to RNA stabilization with Tempus solution at a 1:2 ratio. Asexual parasites (rings, trophozoites, and schizonts) were also counted in the same culture to allow parasite quantification using asexual-stage specific targets.

### DNA and RNA isolation

Total DNA was extracted from three 0.32 cm diameter circles punched from each DBS using the QIAamp 96 Blood Kit (Qiagen, Valencia, CA) per the manufacturer’s instructions and eluted in 50 µl EDTA buffer. RNA was extracted from whole-blood RNA in Tempus using Norgen RNA extraction kit (Norgen Biotek, Thorold, Ontario) and treated with RNase-Free DNase I Kit (Norgen Biotek) to a final elution volume of 50 µl, per manufacturer’s instructions. Extracted RNA samples were assessed for quality and quantity using automated parallel capillary electrophoresis (Fragment Analyzer System, Agilent).

### Real-time quantitative PCR using genomic DNA

To detect the presence of *P. falciparum* genomic DNA isolated from the DBS, primers targeting *P. falciparum varATS* that were originally designed for use in a Taqman-based qPCR assay [21] were adapted for use with the PowerUp SYBR Green Master Mix System (Thermo Fisher Scientific, Waltham, Massachusetts) (Additional file 1: Table S1). Samples were assayed in triplicate in 384-well plates on a QuantStudio 6 Flex Real Time PCR System (Thermo Fisher Scientific, Waltham, Massachusetts) using standard cycling conditions and a melt curve analysis. During assay development, PCR products were Sanger sequenced to verify that wells with a first calculated melt temperature (Tm1) > 71.14 °C contained *varATS* amplicons and wells with Tm1 < 71.14 °C contained primer dimers. Subsequently, the criteria for a *P. falciparum* positive sample were set as having ≥ 2 of 3 replicate wells with a Ct < 39 AND a Tm1 > 71.14 °C. Using these criteria, genomic DNA samples isolated from the blood of 20 of 20 (100%) healthy North American controls with no malaria exposure history were confirmed to be *P. falciparum* negative, and 86 of 87 (98.9%) samples positive by conventional *P. falciparum* 18s rRNA PCR [22, 23] were confirmed as positive with the modified *varATS*-based assay. The one discordant sample had only 1 of 3 replicate wells meeting the Ct and Tm1 criteria. A standard curve of gDNA extracted from serially diluted *P. falciparum*-spiked DBS samples (described above) and no-template negative controls were run on every plate, which allowed for estimation of parasite densities using Ct values.

### Multiplex real-time quantitative reverse transcription PCR

An initial aim of the study was to develop a four-plex real-time quantitative reverse transcription PCR (RT-qPCR) that would detect female gametocytes, male gametocytes, and asexual parasites, as well as a human housekeeping gene glyceraldehyde 3-phosphate dehydrogenase (GAPDH), which served as a control for RNA extraction and relative quantification. The genes *pfs25* [24] and *pfs230p* were used as the female- and male-specific gametocyte targets, respectively. The gene encoding for parasite-infected erythrocyte surface protein (*piesp2*, also called PFE60 and PF3D7_0501200) was chosen based on a transcriptional pattern restricted to asexual blood-stages, particularly trophozoites, in three *P. falciparum* gene expression datasets available on PlasmoDB (http://plasmodb.org) [25–27]. Primers and probes for *pfs25* were adapted from Wampfler et al*.* [28]. Primers and probes for *pfs230p* and *piesp2* were developed de novo using Primer 3 software [29] following standard guidelines for qPCR primer design. All primer and probes are listed in Additional file 1: Table S1.

After generation of complementary DNA (cDNA) from 50 ng RNA for each sample replicate using LunaScript RT Supermix (New England Biolabs) under standard cycling conditions, 20× triplex master mix was prepared from appropriate final concentrations of primers and probes for the three parasite targets and combined with 20× human *GAPDH* master mix (Applied Biosystems), Taqman multiplex master mix (Applied Biosystems), and cDNA to a final reaction volume of 10 μl. Field samples identified as positive for *P. falciparum* by *varATS* qPCR, no reverse transcriptase controls, amplification controls, and tenfold parasite RNA dilution standards were assayed in triplicate in 384-well MicroAMP Optical PCR plates (Applied Biosystems). The targets *pfs25*, *pfs230p*, *piesp2*, and human *GAPDH* were run in a QuantStudio6 Flex qPCR system (Applied Biosystems) with NFQ-MGB Quencher and VIC, FAM, ABY, and JUN reporter dyes, respectively (Additional file 1: Table S1). Mustang Purple was selected as the reference dye. Multiplex assay was run under standard cycling conditions: initial denaturation at 95.0 °C for 20 s (hold stage) followed by 40 cycles of 95.0 °C for 1 s and 60.0 °C for 20 s (PCR stage).

### Statistical analysis

All statistical analyses were performed using R version 4.0.1 (https://www.r-project.org). Sample size estimates were performed using the epiR package. Multiple logistic regression was performed with PCR-confirmed gametocytaemia as the dependent variable and gender, age (in years), recent bed net use, recent travel, log_10_ transformed parasite density, and community as independent variables. Plots were rendered using the ggplot2 package. Statistical tests used to determine significance are indicated in tables and figure legends, and p values < 0.05 were considered significant.

## Results

A total of 1354 participants were enrolled across all communities for this study (Table 1). The RDTs used for point-of-care diagnosis of asymptomatic infections demonstrated a 4.7% false positive rate using *varATS* qPCR as the reference standard. By contrast, microscopy showed no false positives. Given this, RDT data was not used for subsequent analyses. The prevalence of asymptomatic infections was greatest in Ajigo, followed by Webuye, regardless of diagnostic modality (Table 1). Only two asymptomatic infections were detected by PCR in Kapsisywa, and no infections were detected in Kipsamoite. Parasite densities were not statistically different across the four communities (Table 1) and did appear to vary with infection prevalence (Additional file 1: Fig. S1). The use of ITNs was higher in Ajigo and Webuye relative to Kapsisywa and Kipsamoite (Table 1). Given the similarities in prevalence of asymptomatic infections in the two highlands communities Kapsisywa and Kipsamoite, they were treated as a single community “Kap-Kip” for all subsequent analyses.

**Table 1:** Participant characteristics

	N	Ajigo	Webuye	Kapsisywa	Kipsamoite	Test statistic
		(N = 235)	(N = 210)	(N = 458)	(N = 451)	
Female gender (%)	1354	137/235 (58.3)	131/210 (62.4)	209/458 (45.6)	235/451 (52.1)	χ^2^ = 20.08, P < 0.01^2^
Age	1354					F = 3.90, P = 0.01^1^
Median age in years (IQR)		13 (7–19)	12.0 (6.9–23.3)	11 (6–17)	13 (7–23)	
Range		3–80	0–70	1–84	1–74	
Rapid diagnostic test (% positive, 95% CI)	1354	124/235 (52.8, 46.2–59.3)	29/210 (13.8, 9.6–19.4)	6/458 (1.31, 0.53–2.98)	3/451 (0.67, 0.17–2.10)	χ^2^ = 476.13, P < 0.01^2^
Asexual microscopy (% positive, 95% CI)	1354	63/235 (26.8, 21.4–33.0)	10/210 (4.76, 2.44–8.84)	0/458 (0.0)	0/451 (0.0)	χ^2^ = 263.29, P < 0.01^2^
Microscopy positive	73					F = 1.54, P = 0.22^3^
Median parasites/μl (IQR)		733 (218–3610)	3970 (212–8190)	–	–	
Range		60–51,627	80–9800	–	–	
*varATS* qPCR (% positive, 95% CI)	1354	99/235 (42.1, 35.8–48.7)	21/210 (10.0, 6.44–15.1)	2/458 (0.437, 0.0757–1.75)	0/451 (0.00)	χ^2^ = 400.35, P < 0.01^2^
*varATS* qPCR (+)	122					F = 0.34, P = 0.80^1^
Median parasites/μl (IQR)		117 (13–1100)	32.6 (4.8–2250)	243 (71–414)	–	
Range		0–90,600	0.2–44,200	71–414	–	
Recent ITN use (%)		196/235 (83.4)	189/210 (90.0)	287/458 (62.7)	301/451 (66.7)	χ^2^ = 74.66, P < 0.01^2^

*N* number of non-missing value, *CI* confidence interval, *IQR* interquartile range, *ITN* insecticide-treated bed net

^1^Kruskal–Wallis

^2^Pearson

^3^Wilcoxon

**Fig. 1:**
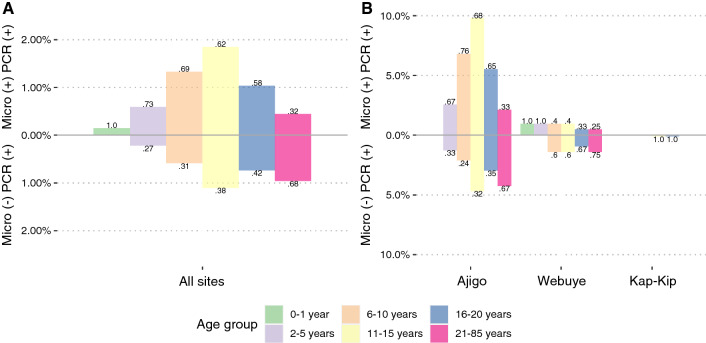
Prevalence of asymptomatic *P. falciparum* infections. Iceberg plot showing microscopy-detectable (above x axis) and sub-microscopic, PCR-detectable (below x axis) *P. falciparum* infections as a proportion of all individuals tested stratified by age group at **A** all sites or **B** by community. Numbers above and below each bar represent proportion of microscopy-detectable and sub-microscopic infections in each age group, respectively

Across all communities, children aged 11–15 years account for the greatest prevalence of sub-microscopic (1.1%; 95% confidence interval [CI], 0.65% to 1.9%) and total PCR-detectable (3.0%; 95% CI 2.2% to 4.0%) asymptomatic infections (Fig. 1A). In contrast to the younger age groups, in which the majority of infections are detectable by microscopy, 68% of asymptomatically infected adults > 21 years of age have sub-microscopic parasitaemia (Fig. 1A), which suggests acquisition of blood-stage immunity [30, 31]. Similar findings were observed in Ajigo and Webuye when asymptomatic infection prevalence was separated by community with the notable observation that in individuals aged 6–20 years the majority of asymptomatic infections in Ajigo were detectable by microscopy, whereas in Webuye, the majority of infections in this age range were sub-microscopic (Fig. 1B). Multiple logistic regression confirmed that being a child aged 11–15 years and residence in the high-transmission settting of Ajigo were independent predictors for *varATS*-detectable parasitaemia after adjusting for gender, ITN use, and recent travel (Additional file 1: Table S2).

For individuals identified as parasitemic by *varATS* qPCR, both asexual parasite densities and sexual parasite densities were quantified within the same sample by four-plex RT-qPCR (see “Methods”). Female and male gametocytes were quantified using qRT-PCR targeting *pfs25* and *pfs230p*, respectively, and identified 122 of 1354 (9.0%; 95% CI 7.6% to 10.7%) as having gametocytaemia based on the quantifiable expression of either gene. Among individuals with *varATS*-detectable parasitaemia (n = 122), there were no significant differences in gender distribution, age, use of ITNs, history of recent travel, or site distribution between those with and without gametocytes by univariate analysis (Table 2). To determine the relationship between gametocytaemia and total parasitaemia, female and male gametocyte densities estimated by RT-qPCR were plotted against corresponding estimated asexual parasite densities. The initial plan was to use parasite densities estimated from *piesp2* Ct values obtained from the same multiplex RT-qPCR assay, which would maintain internal consistency for each sample. However, *piesp2*-derived parasite densities demonstrated poorer correlation with microscopy-determined parasite densities in patent infections and less sensitivity than gDNA-based detection using *varATS* (Additional file 1: Fig. S2). Thus, total parasite densities derived from the *varATS*-based assay were used to approximate asexual parasite densities for the remainder of the study. Inclusion of *varATS*-estimated total parasite densities in a multiple logistic regression model revealed a decreased risk of gametocytaemia in the lower transmission communities relative to the high-transmission community of Ajigo and among individuals who reported recent travel (Table 3). As expected, increased total parasite densities greatly increased the likelihood of gametocytaemia independent of site (Table 3).

**Table 2:** Comparison of gametocyte negative and gametocyte positive individuals

	Gametocyte (−)	Gametocyte (+)	Test statistic
	(n = 20)	(n = 102)	
Female gender	11/20 (55.0%)	53/102 (52.0%)	χ2 = 0.06, P = 0.80^1^
Age			F statistic = 0.56, P = 0.45^2^
Median (interquartile range)	14.0 (7.4–28.2)	13 (9–16)	
Range	3–62	1–80	
No recent ITN use	3/20 (15.0%)	21/102 (20.6%)	χ2 = 0.33, P = 0.57^1^
No recent travel	14/20 (70.0%)	88/102 (86.3%)	χ2 = 3.23, P = 0.07^1^
Site (% positive, 95% CI)			χ2 = 1.72, P = 0.42^1^
Ajigo	16/20 (80.0, 55.7–93.4)	83/102 (81.4, 72.2–88.1)	
Webuye	3/20 (15.0, 3.96–38.9)	18/102 (17.6, 11.1–26.7)	
Kap-Kip	1/20 (5.0, 0.26–27)	1/102 (0.98, 0.051–6.1)	

Among individuals with parasitaemia confirmed by *varATS* qPCR

*ITN* insecticide-treated bed net, *CI* confidence interval

^1^Pearson χ^2^

^2^Wilcoxon

**Table 3:** Multiple logistic regression to assess the risk of gametocytemia

Variable	OR	95% CI	p-value	q-value
Female	1.00	0.55, 1.85	> 0.9	> 0.9
Age	1.01	0.99, 1.03	0.2	0.3
Recent ITN use (reference: no use)	0.66	0.33, 1.35	0.2	0.3
Recent travel (reference: no travel)	0.34	0.12, 0.87	0.037	0.073
log_10_ (parasite/μl + 1)^a^	9.40	6.11, 15.5	< 0.001	< 0.001
Site (reference: Ajigo)				
Webuye or Kap-Kip	0.10	0.05, 0.18	< 0.001	< 0.001

For site, Kap-Kip and Webuye were combined given there were only 2 infected individuals in Kap-Kip

*OR* odds ratio, *CI* confidence interval, *ITN* insecticide-treated bed net

^a^Parasite densities were estimated from *varATS* qPCR Ct values using standard curves (see “Methods”)

**Fig. 2:**
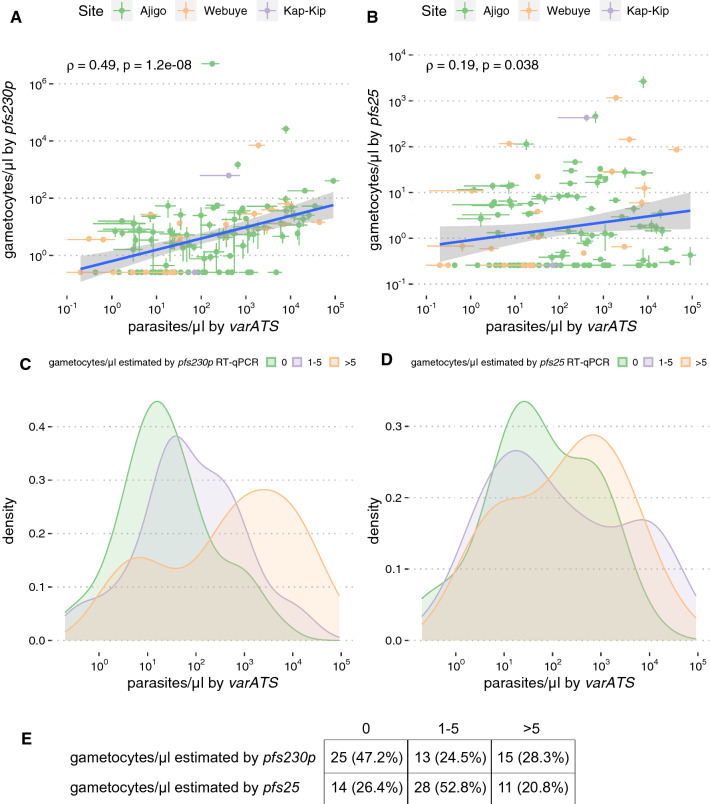
Relationship between total parasitaemia and gametocytemia. Spearman’s rank correlations between parasites/μl estimated by *varATS* and gametocytes/μl estimated by **A** *pfs230p* or **B** *pfs25*. Density plots of parasites/μl by number of gametocytes/μl estimated by **C** *pfs230p* or **D** *pfs25*. **E** Numbers (row proportions) of *varATS*-PCR positive individuals by number of gametocytes/μl

In general, both male and female gametocytaemia increased with increasing total parasitaemia (Fig. 2A, B). However, some individuals with very low total parasite densities were noted to have unexpectedly high gametocyte densities. Indeed, individuals with > 5 gametocytes/µl were bimodally distributed across a wide range of total parasitemia, which was more marked for *pfs230p* (Fig. 2C, D). Among individuals with low-density infections (total parasite densities < 40 parasites/μl), 28.3% (95% CI 17.2% to 42.6%) had > 5 gametocytes/μl estimated by *pfs230p*, and 20.8% (95% CI 11.3% to 34.5%) had > 5 gametocytes/μl estimated by *pfs25* (Fig. 2E).

**Fig. 3:**
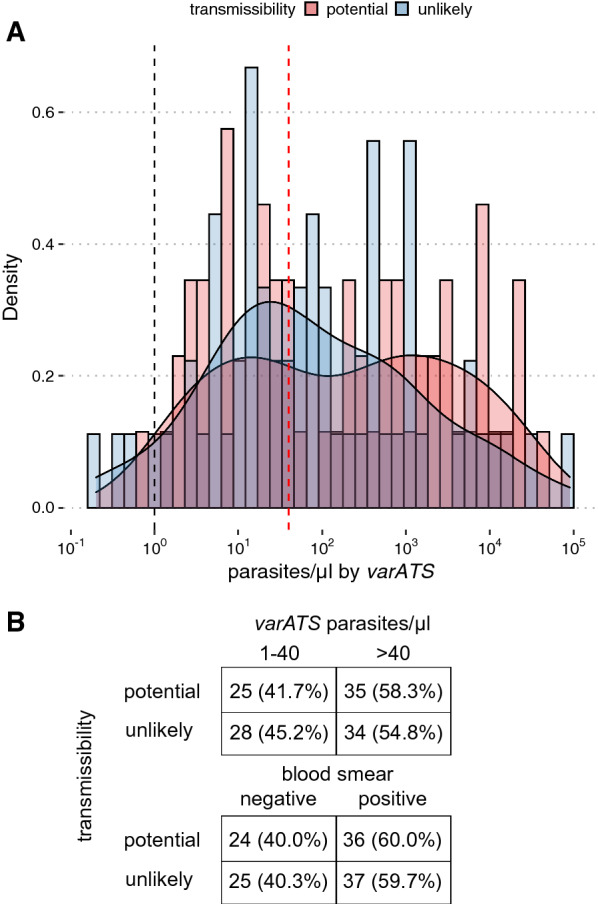
Distribution of individuals with potential for onward transmission. **A** Density plots by potential for onward transmission. Limit of detection for conventional molecular diagnostics and microscopy are shown as dashed black and red vertical lines, respectively. **B** Numbers (row proportions) of potential and unlikely transmitters by parasite density and blood smear positivity. Individuals with potential for onward transmission are defined in as having at least 1.25 female and four male gametocytes per 2.5 µl of blood, which are increased thresholds adapted from a prior study [12] to account for sex-specific gametocytaemia overestimation

A substantial proportion (41.7%; 95% CI 29.3% to 55.1%) of individuals with potential for onward transmission, defined in our study as having at least 1.25 female and four male gametocytes per 2.5 µl of blood (thresholds adapted from a prior study [12] to account for sex-specific gametocytaemia overestimation), had qPCR-estimated parasite densities above the detectable limit of conventional, 18s ribosomal RNA-based nested PCR (1 parasite per µl) [22] and below the limit of detection of microscopy (40 parasites per µl), which corresponded well to the actual proportion potential transmitters with submicroscopic infections (40.0%; 95% CI 27.8% to 53.5%; Fig. 3A, B).

## Discussion

The current descriptive study provides a cross-sectional assessment of asymptomatic *P. falciparum* infections of three communities in western Kenya with differing malaria transmission intensities from August to September 2016. [12]. Similar to prior assessments, transmission intensity remained low in Kap-Kip [18, 32], where asymptomatic *P. falciparum* parasitaemia was rarely detected by *varATS* qPCR (0.22% prevalence). High malaria transmission was observed in Ajigo, where 42% of individuals had asymptomatic parasitaemia. Webuye demonstrated moderate transmission with 10% prevalence of asymptomatic parasitaemia, which is lower than what has been previously described at this site [33, 34], possibly reflecting micro-heterogeneity or seasonal differences, as the current study was performed during months when rainfall is historically lower in western Kenya.

The substantial reservoir observed among 6–15 year old children in Ajigo and Webuye is consistent with a prior study in the Kakamega district of western Kenya that showed PCR-confirmed asymptomatic *P. falciparum* infections were more prevalent in younger children age 5–14 years (~ 34%) relative to older children > 14 years (~ 9%) [35]. However, this observation contrasts with a study conducted in a high transmission area (Suba district) that demonstrated the prevalence of blood-smear positive asymptomatic infections was greater in young children < 5 years (74%) compared to older children (30–50%) [36]. The differences in relative contribution to the asymptomatic infectious reservoir by age groups may be attributable to intense malaria transmission in Suba, where clinical immunity may be acquired more rapidly, and differences in assay sensitivity.

To determine whether sensitive molecular assays can sufficiently detect the majority of individuals carrying low-density *P. falciparum* infections who are also potentially capable of onward transmission, the relationship between asexual parasitaemia and gametocytaemia was assessed. The initial goal was to correlate gametocyte densities with asexual parasite densities using a multiplex RT-qPCR that would contain targets specific to female gametocytes, male gametocytes, and asexual blood-stage parasites in a single assay, which would facilitate comparisons as this strategy eliminates both within subject differences in template preparation and assay variability. However, parasite densities determined using the chosen asexual-specific target *piesp2*, which encodes for parasite-infected erythrocyte surface protein and previously shown to be maximally transcribed in the trophozoite stage in laboratory isolates [25–27, 37], showed weaker correlation with microscopy-determined parasite density than densities derived from *varATS* qPCR using gDNA (Additional file 1: Fig. S2). The weaker correlation for *piesp2* could be due to lower *piesp2* expression in ring stages, which had previously been thought to be the predominant asexual form of *P. falciparum* found in peripheral circulation. However, a recent study in Mali revealed that more developed trophozoite stages were commonly found in asymptomatic *P. falciparum* infections [38]. Expression of *piesp2* could also vary among the field isolates, perhaps due to differential transcriptional regulation related to precise stage at the time of collection or host immune pressure. Although speculative, these potential explanations are intriguing given that antibodies against PIESP2 associate with protection from malaria [39], and PIESP2 has recently been observed to bind to brain microvascular endothelial cells in vitro to induce an inflammatory response [40]. The current data, combined with these prior findings, suggest that *piesp2* is a poor target for quantifying asexual parasite densities.

Nevertheless, using *varATS*-derived parasite densities, asexual parasitaemia and residence in a high-transmission setting independently predicted gametocytaemia, consistent with a recent longitudinal analysis of gametocyte carriage in Kilifi, Kenya [41]. On the surface, this might suggest that treating high-density infections in high-transmission settings, especially with ACT that is highly effective against early stage gametocytes (e.g. artemether–lumefantrine or artesunate/mefloquine) [42], would contribute to the overall reduction of gametocyte carriage. However, such a strategy neglects the potential contribution of sub-microscopic infections. The observation that a sizable proportion of low-density infections (< 40 parasites/μl) had estimated gametocyte densities that would favour onward transmission is also consistent with prior studies that demonstrated a considerable sub-microscopic infectious reservoir [4, 10, 43, 44]. Although the presence of gametocytaemia was only determined among individuals who were parasitaemic by *varATS* qPCR, which had a limit of detection of ~ 0.4 parasites/μl using dried blood spots, the proportion of individuals with potential for onward transmission drops off below 10 parasite/μl. This finding is in line with recent studies suggesting that mosquito infectivity occurs primarily when parasitaemia is > 1 parasite/μl [12, 45], which is the limit of detection of standard molecular diagnostics. Taken together, the main implication is that ultra-sensitive molecular diagnostics capable of detecting infections < 1 parasite/μl may not be necessary to achieve significant reductions in malaria transmission using a screen-and-treat strategy. However, experimental studies are needed to definitively determine whether the low-density infections in communities such as Ajigo and Webuye contribute significantly to malaria transmission.

There are several limitations to the current study. Although the study excluded individuals who endorsed symptoms of acute illness at the time of enrollment and sample collection, clinical or laboratory examinations that may have uncovered a more subacute or indolent disease process beyond self-reported symptoms were not performed. No short-term follow-up was conducted to assess whether asymptomatic individuals progressed to symptomatic malaria as other studies have done [46, 47]. By including potentially pre-symptomatic individuals, the prevalence of true asymptomatic infections may have been overestimated. Additionally, the cross-sectional study design provides only a snapshot of infection prevalence in these communities during the relatively dry season and the current findings may not be generalizable to the rainy season when malaria transmission is more intense. Gametocyte densities were estimated using molecular quantification of male and female gametocyte-specific gene expression as a surrogate of potential for onward transmission and did not directly measure mosquito infectivity using direct or indirect feeding assays. Such a surrogate based solely on gametocyte density ignores the relative contribution of anti-gametocyte immunity in reducing malaria transmission [48]. Although male and female gametocyte targets were used for gametocyte quantification, sex was not differentiated when determining gametocyte densities by microscopy for standard curves, which would lead to overestimates of sex-specific gametocytaemia. This is especially true for male-specific gametocytaemia given that natural infections are biased towards females, with 3–5 times more females [49]. However, no assessments using sex ratio were conducted in this study. Furthermore, gametocytaemia overestimates would affect all samples consistently and thus would not affect the ranked correlation analyses. Importantly, in determining the number of individuals capable of onward transmission, the effect of overestimating sex-specific gametocytaemia was reduced by using higher thresholds for minimum male and female gametocyte densities.

## Conclusion

In summary, this cross-sectional survey of the prevalence and densities of *P. falciparum* infections among asymptomatic individuals in western Kenya provides an assessment of the relationship between parasitaemia and gametocytaemia in three communities with different transmission intensities. These data provide evidence of a substantial sub-patent infectious reservoir among asymptomatic carriers in these communities and supports prior findings that conventional molecular diagnostics may be capable of detecting the vast majority of infections capable of onward transmission [12]. Development of field-deployable molecular diagnostics to be used for the identification and treatment of asymptomatic carriers of *P. falciparum* could accelerate progress towards malaria elimination by reducing the infectious reservoir.

## Supplementary Information

**Additional file 1: Table S1.** Supplementary tables and figures.

## Data Availability

Data and material used in this study will be made available upon request.

## Abbreviations

cDNA: Complementary deoxyribonucleic acid
CI: Confidence interval
DBS: Dried blood spot
EDTA: Ethylenediaminetetraacetic acid
gDNA: Genomic deoxyribonucleic acid
ITN: Insecticide-treated net
MR4: Malaria Research and Reference Reagent Resource Center
PCR: Polymerase chain reaction
RDT: Rapid diagnostic test
RNA: Ribonucleic acid
RT-qPCR: Real-time quantitative reverse transcription PCR
