# Supplementary material for: The prevalence and density of asymptomatic Plasmodium falciparum infections among children and adults in three communities of western Kenya
Source: Malar J. 2021 Sep 17;20:371. doi: 10.1186/s12936-021-03905-w (PMC8447531; doi:10.1186/s12936-021-03905-w)
Supplement: Supplementary file 1 — Additional file 1: Table S1. Supplementary tables and figures. [file 12936_2021_3905_MOESM1_ESM.pdf]

Additional file 1:

Title: The prevalence and density of asymptomatic *Plasmodium falciparum* infections among children and adults in three communities of western Kenya

Authors: Christina Salgado[1], George Ayodo[3,4], Michael D. Macklin[1], Meetha P. Gould[5], Srinivas Nallandhighal[1], Eliud O. Odhiambo[2,3], Andrew Obala[6], Wendy P. O'Meara[7], Chandy C. John[2], Tuan M. Tran[1,2]

Affiliations:

1. Division of Infectious Diseases, Department of Medicine, Indiana University School of Medicine, Indianapolis, Indiana, USA
2. Ryan White Center for Pediatric Infectious Diseases and Global Health, Department of Pediatrics, Indiana University School of Medicine, Indianapolis, Indiana, USA
3. Center for Global Health Research, Kenya Medical Research Institute, Kisumu, Kenya
4. Jaramogi Oginga Odinga University of Science and Technology, Bondo, Kenya.
5. Reserve Analytics, LLP, Cleveland, Ohio USA
6. School of Medicine, Moi University College of Health Sciences, Eldoret, Kenya
7. Duke Global Health Institute, Duke University, Durham, North Carolina, USA.

Table S1: Primer and Probe Sequences

assay	primer/probe name	sequence	reference
varATS RT qPCR	varATS For	5' CCCATACACAACCAAYTGGA 3'	Hofmann et al. 2015 [21]
	varATS Rev	5' TTCGCACATATCTCTATGTCTATCT 3'	Hofmann et al. 2015 [21]
multiplex RT-qPCR	Pfs230p_2_F	5' TTGCCTGTTACCTTATTGTCAAG 3'	
	Pfs230p_2_R	5' CCGTCGACATCTTCAGCAT 3'	
	Pfs230p_2_IN	6FAM-CCAGGCTGATGAAGACATCGGG-QSY	
	Pfs25 For	5' GAAATCCCGTTTCATACGCTTG 3'	Wampfler et al. 2013 [28]
	qPCR Pfs25 Rev	5’ AGTTTTAACAGGATTGCTTGTATCTAA 3'	Wampfler et al. 2013 [28]
	qPCR Pfs25_probe	VIC-TGTAAGAATGTAACTTGTGGTAAC-QSY	adapted from Wampfler et al. 2013 [28]
	Pf3D7_ESP_F	5' TAGGTCAAGCACCTCCAGAA 3'	
	Pf3D7_ESP_R	5' TCATTAGGGTCAAGTGCGTAA 3'	
	Pf3D7_ESP_IN	ABY-TGGTTCATTTCCCCAAGCAGGTG-QSY	

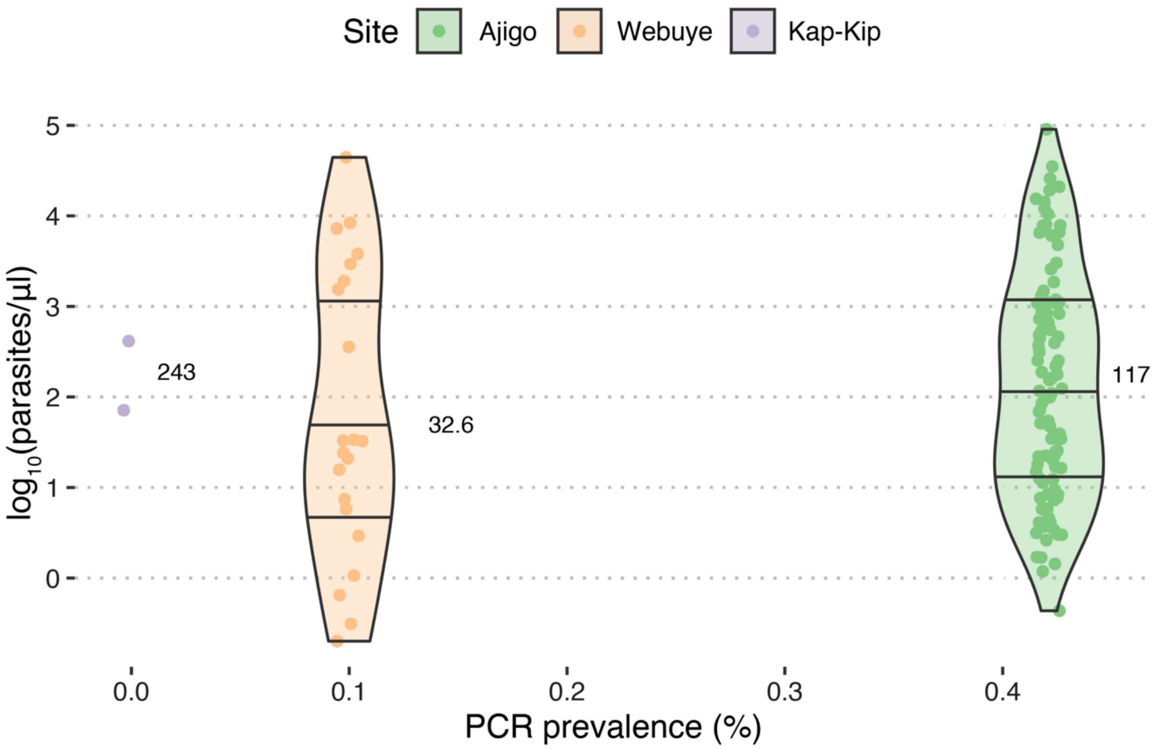

Figure S1. Relationship between parasite density and prevalence of PCR-detective asymptomatic P. falciparum infections by community. Median parasite density indicated to right of each violin plot.

Table S2: **Multiple logistic regression to assess the risk of *varATS*+ parasitemia**

**Variable**	**OR**	**95% CI**	**p-value**	**q-value**
Female gender	0.72	0.45, 1.16	0.2	0.3
Age (reference: 11-15 years)				
0-1 year	1.45	0.20, 6.67	0.7	0.7
2-5 years	0.18	0.08, 0.39	<0.001	**<0.001**
6-10 years	0.46	0.24, 0.90	0.024	**0.048**
16-20 years	0.72	0.35, 1.49	0.4	0.5
21-85 years	0.30	0.15, 0.61	0.001	**0.003**
Recent ITN use (reference: no use)	0.69	0.37, 1.31	0.2	0.4
Recent travel (reference: no travel)	0.83	0.44, 1.54	0.6	0.6
Site (reference: Ajigo)				
Webuye	0.14	0.08, 0.24	<0.001	**<0.001**
Kap-Kip	0.00	0.00, 0.01	<0.001	**<0.001**

OR = odds ratio; CI = confidence interval; ITN = insecticide-treated bed net

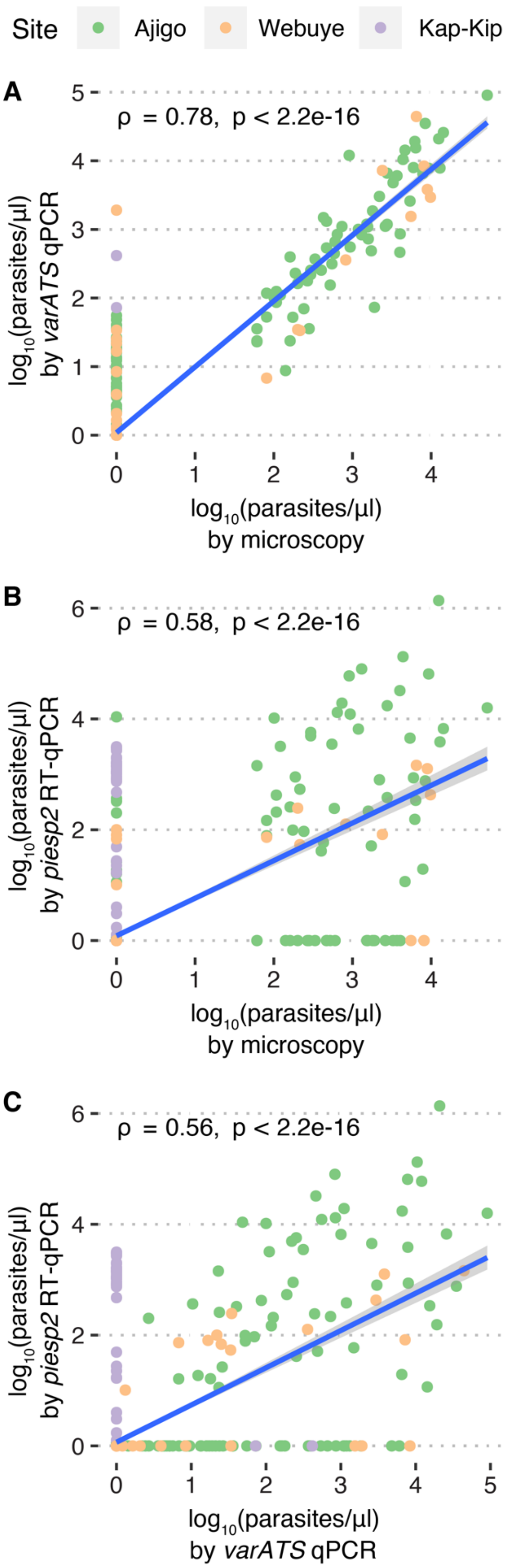

Figure S2. Correlation between parasite quantification assays. Spearman's rank correlation coefficients (ρ) were determined between parasite densities estimated by **(A)** *varATS* qPCR and microscopy, **(B)** *piesp2* RT-qPCR and microscopy, and **(C)** *piesp2* RT-qPCR and microscopy. Blue lines are best linear fit lines with 95% confidence intervals.
